# Assessing the potential for outcome reporting bias in a review: a tutorial

**DOI:** 10.1186/1745-6215-11-52

**Published:** 2010-05-12

**Authors:** Kerry Dwan, Carrol Gamble, Ruwanthi Kolamunnage-Dona, Shabana Mohammed, Colin Powell, Paula R Williamson

**Affiliations:** 1 Centre for Medical Statistics and Health Evaluation, University of Liverpool, UK; 2 Medical Care Research Unit, University of Sheffield, UK; 3 University Department of Paediatrics, School of Medicine, Cardiff University, Children's Hospital for Wales, Cardiff, UK

## Abstract

**Background:**

Outcome reporting bias (ORB) occurs when variables are selected for publication based on their results. This can impact upon the results of a meta-analysis, biasing the pooled treatment effect estimate.

The aim of this paper is to show how to assess a systematic review and corresponding trial reports for ORB using an example review of intravenous and nebulised magnesium in the treatment of asthma.

**Methods:**

The review was assessed for ORB by 1) checking the reasons, when available, for excluding studies to ensure that no studies were excluded because they did not report the outcomes of interest in the review; 2) assessing the eligible studies as to whether the review outcomes of interest were reported. Each study was classified using a system developed in the ORBIT (Outcome Reporting Bias In Trials) project to indicate whether ORB was suspected and a reason for the suspicion. Authors of trials that did not report the outcomes of interest were contacted for information. A sensitivity analysis was performed to assess the robustness of the conclusions of the review to this potential source of bias.

**Results:**

Twenty-four studies were included in the review; two studies had been excluded for not reporting either of the two outcomes of interest. Six included studies did not report hospital admission and two did not report pulmonary function. There was high suspicion of outcome reporting bias in four studies. Results from the sensitivity analysis indicate that review conclusions were not overturned.

**Conclusion:**

This paper demonstrates, with the example of the magnesium review, how to assess a review for outcome reporting bias. A review should not exclude studies if they have not reported the outcomes of interest and should consider the potential for outcome reporting bias in all included studies.

## Background

Over the past decade, evidence-based medicine and evidence-based health policy have become dominant themes in clinical and health services research [1]. A systematic review is a method used to review research literature and summarise evidence from multiple studies that fit pre-specified eligibility criteria in order to answer a specific research question [2]. Systematic reviews are important as they can identify areas where the evidence that is available is insufficient and new trials are required [3].

Empirical research in randomised controlled trials (RCTs) provides strong evidence of an association between studies that report positive or significant results (P < 0.05) and publication; with studies that report positive or significant results being more likely to be published [4]. Such bias, termed study publication bias, is well recognised as a potential threat to the validity of any meta-analysis and can make the readily available evidence unreliable for decision making [5,6].

Outcome reporting bias (ORB) has been defined as the selection on the basis of the results of a subset of the original outcomes recorded for inclusion in publication of trials [7]. Up until recently ORB has received less attention than study publication bias. A recent review of empirical research provides strong evidence that outcomes that are statistically significant have higher odds of being fully reported (range of odds ratios: 2.2 to 4.7) [4]. Sensitivity analyses have been suggested to assess the robustness of the conclusions of a meta-analysis to ORB [6,8].

Cochrane reviews now include an assessment of the risk of bias within each included study [9]. There are six included domains: sequence generation; allocation concealment; blinding; incomplete outcome data; selective outcome reporting and other sources of bias. For 'selective outcome reporting,' the reviewers must state how the possibility of selective outcome reporting was examined and what was found.

The prevalence and impact of outcome reporting bias in an unselected cohort of systematic reviews has recently been described [10] (Appendix 1). It was found that a third of Cochrane reviews contained at least one trial with high ORB suspicion for the review primary outcome. Within this work, a nine point classification system was developed to assess the risk of ORB within trial reports.

The aim of this paper is to illustrate how to assess a systematic review for ORB, how to classify suspicion of ORB within the trials using the nine point classification system and how to undertake a sensitivity analysis to assess the robustness of the conclusions of a meta-analysis to this potential source of bias along with study publication bias.

## Steps to consider in examining potential for ORB

### Assessing a review

The first step when assessing a review for ORB is to check the reasons for excluding studies to ensure that no studies were excluded because they did not report the outcomes of interest in the review (Appendix 2). If a trial report does not give results for, or mention certain outcomes this does not necessarily mean that they were not measured or analysed. Cochrane reviews routinely provide a list of excluded studies with reasons for their exclusion. When the review is not a Cochrane review and this information is not provided within the publication it may be necessary to contact the review authors. The second step is to check the studies that were included in the review as to whether they report none, one or all outcomes the reviewers are interested in.

The third step is to obtain the trial reports of those studies that were listed in the review (or through contact with the reviewer if a list was not available) as excluded because they did not report on any of the outcomes of interest and the eligible studies which did not report on one or more of the outcomes of interest. Using the trial reports a matrix is constructed, with the outcomes of interest in the review and those reported in the trial reports listed in columns and the different studies listed in the rows (Table 1). The reason other trial outcomes are looked at is that in some cases outcomes may be structurally related so that if one outcome was reported, it is known that the other must have been measured. For example, if length of hospital stay had been reported then hospital admission must have been noted. Also, some outcomes are often measured routinely together so that if one outcome is reported but not the other this may raise suspicions that selective reporting has occurred e.g. systolic and diastolic blood pressure.

**Table 1:** Outcome matrix

Trial ID	Review primary outcomes		Trial outcomes								
	
	Pulmonary function	Hospital admission	Blood pressure	Severity score	Respiratory score	Calcium/potassium/magnesium	Respiratory frequency or rate	Hospital stay/length of hospital stay	Heart rate	Side effects	Other outcomes
Dadhich, 2003 [17]	✘	✘	✘	✘	✘	✘	✘	✘	✘	○	Bronchodilating effects

Santana, 2001 [18] (children)	✘	✘	○	✘	✘	✔	✔	✔	✔	✔	arterial blood gasses, oxygen therapy, ph, nebulisations, acidosis

Bijani, 2002 [22] (adults)	✔	✘	✘	✘	✘	✘	✘	✘	✘	✘	Number of breathing, diaphoresis, cyanosis, Using respiratory access muscles, clinical asthma score

Bessmertny, 2002 [21] (adults)	✔	✘	○	✘	✘	○	○	✘	○	✔	oxygen saturation

Gurkan, 1999 [24] (children)	✔	✘	○	✘	✘	✘	✘	✘	○	○	Nebulisations, clinical asthma score

Devi, 1997 [23] (children)	✔	✘	✘	✘	✘	✘	✘	✘	✔	✔	Clinical asthma score, oxygen saturation, pulsus paradoxus

Tiffany, 1993 [26] (adults)	✔	✘	✘	✘	✘	✔	✘	✘	✘	✘	✘

Meral, 1996 [25] (children)	✔	✘	○	✘	✔	✘	○	✘	○	✔	✘

Boonyavorakul, 2000 [19] (adults)	✘	✔	✘	✔	✘	✘	✘	✘	✘	✘	✘

Scarfone, 2000 [20] (children)	✘	✔	✘	✘	✘	✘	✘	✘	✘	✘	Pulmonary index score, oxygen saturation

^✔ ^indicates full reporting of results for treatment comparison of interest

^✘ ^indicates no reporting

**○ **indicates partial reporting (i.e. only the *p-value *is given for the comparison)

### Trial assessment using the classifications

For eligible studies not reporting the outcomes of interest, it is important to assess the likelihood that the outcomes had been measured and selectively not reported. The fourth step is to complete the matrix constructed in step three by applying the ORBIT nine point classification system.

Each study is given a classification (see Table 2) along with a reason, using verbatim trial report text whenever appropriate to support the chosen classification. The classification is determined by comparing the methods to the results section of the trial report, by looking at which other outcomes were measured and reported and accounting for knowledge of the clinical area. This should be completed by at least two people independently and differences should be discussed to agree on an overall classification to determine whether there is a high or low risk of outcome reporting bias.

**Table 2:** The ORBIT classification system for missing or incomplete outcome reporting [10].

Classification	Description	Level of reporting	Level of suspicion of ORB
*Clear that the outcome was measured and analysed*			

**A**	States outcome analysed but only reported that result not significant (typically stating p-value > 0.05).	Partial	High risk

**B**	States outcome analysed but only reported that result significant (typically stating p-value < 0.05).	Partial	Low risk

**C**	States outcome analysed but insufficient data presented to be included in meta-analysis or to be considered to be fully tabulated.	Partial	Low risk

**D**	States outcome analysed but no results reported.	None	High risk

*Clear that the outcome was measured*			

**E**	Clear that outcome was measured but not necessarily analysed.	None	High risk

**F**	Clear that outcome was measured but not necessarily analysed.	None	Low risk

*Unclear that the outcome was measured*			

**G**	Not mentioned but clinical judgment says likely to have been measured and analysed.	None	High risk

**H**	Not mentioned but clinical judgment says unlikely to have been measured.	None	Low risk

*Clear that the outcome was NOT measured*			

**I**	Clear that outcome was not measured.	N/A	No risk

### Contacting authors

After the matrix has been completed in step four, step five involves an attempt to contact the trialists from the trials included in the review that did not report the outcomes of interest. The purpose of this contact is to clarify whether the chosen classification is correct and when possible to obtain data to include in an updated meta-analysis. However, in the process of conducting a review, it could be argued that this step should be taken straight after identifying studies not reporting the outcome of interest.

## Sensitivity analysis for assessing the impact of ORB

The purpose of the sensitivity analysis in step six is to determine whether the conclusions of the meta-analysis are robust to the assumption that selective reporting has occurred. A method proposed to consider the potential effect of study publication bias has been adapted for ORB [8] and may be used to simultaneously consider robustness to both ORB and study publication bias. The formula used in this sensitivity analysis is provided as a worked example in appendix 3. The sensitivity analysis provides a treatment effect estimate and confidence interval which should be compared against the original results to consider the robustness of that result.

The method is applied when the number of eligible studies not reporting the outcome of interest is known, additional information obtained from trialists may also be utilised. For example the trialists may confirm that the outcome was not measured eliminating that study from suspicion of ORB. The sensitivity analysis can also be calculated for an additional number of unpublished or unobserved studies to see how many would be required to overturn the conclusions of the meta-analysis. Then plausibility of this number can then be considered.

Reviewers may wish to obtain statistical advice regarding application of the proposed sensitivity analysis and the interpretation of the results. However, this sensitivity analysis can be conducted in excel and an excel file can be obtained from the first author on request.

## Example

The systematic review '*Intravenous and nebulised magnesium sulphate for acute asthma' *[11] was assessed. Interest in this review arose as three of the co-authors (RKD, CP and PRW) are involved in the HTA-funded MAGnesium NEbuliser Treatment In Children (MAGNETIC) study (HTA 05/503/10, http://www.hta.ac.uk/1615). This review is clinically important as asthma affects 5.2 million people in the UK, including 1.1 million children [12], and is responsible for around 63 000 hospital admissions per year [13]. Magnesium sulphate has been suggested as an alternative treatment option in patients resistant to standard asthma therapy [11], either in intravenous or nebulised dosage form.

Pulmonary function tests (PFTs) and hospital admission are clinically relevant to acute asthma [14], and consequently are the primary outcomes of the review. Peak expiratory flow rate (PEFR) and forced expiratory volume in one second (FEV1) are common pulmonary function tests [15]. This review includes both adults and children however it is difficult to obtain PEFR measurements in children with acute asthma, so trials involving children may not have measured this outcome [16]. Meta-analyses were conducted in this review, subgrouped by trials with adults and children, for studies using intravenous and nebulised magnesium compared with placebo (Table 3).

**Table 3:** Results of meta-analyses and sensitivity analyses.

Outcome	Review results	Number of studies suspected of ORB	Number of participants missing from the meta-analysis (%)	Sensitivity analysis results	
				
				ORB alone	Study publication bias *
**Intravenous Magnesium: children**					

Hospital admission	RR 0.69 (95% CI 0.53, 0.90) from 3 studies, I^2 ^= 17.7%, RE. Favours intervention	3 [18,23,24]	117 (50%)	RR 0.76 (95% CI: 0.58, 0.99) for one study as trialists confirmed that for the other two it was not measured.	1

Pulmonary function	SMD 1.94 (95% CI: 0.80, 3.08) from 4 studies, I^2 ^= 84.4%, RE. Favours intervention	2 [18,20]	104 (45%)	NA: both studies given H classifications and this was confirmed by trialists	4

**Intravenous Magnesium: adults**					

Hospital admission	RR 0.87 (95% CI 0.70, 1.08) from 8 studies, I^2^- = 30%, RE. Favours intervention	2 [22,26]	129 (14%)	NA: confirmed by trialists that this was not measured	4

Pulmonary function	SMD 0.25 (95% CI -0.01, 0.51) from 9 studies, I^2 ^= 70.6%, RE. Favours intervention	1 [19]	33 (3%)	NA: results from one study obtained and included in an updated meta-analysis	
	Updated SMD 0.24, (95% CI 0, 0.48) from 10 studies, RE. I^2 ^= 67%				7

**Nebulised Magnesium: children**					

Hospital admission	RR 2 (95% CI 0.19, 20.93) from 1 study, RE. Favours control	1 [25]	40 (39%)	NA: confirmed by the trialists that this was not measured	1

Pulmonary function	SMD -0.26 (95% CI -1.49, 0.98) from 2 studies, I^2 ^= 88.9%, RE. Favours control	0	0	NA: all eligible studies reported on this outcome	1

**Nebulised Magnesium: adults**					

Hospital admission	RR 0.68 (95% CI 0.46, 1.02) from 6 studies, I^2 ^= 0, RE. Favours intervention	1 [21]	74 (17%)	RR 0.76 (95% CI 0.51, 1.13)	8

Pulmonary function	SMD 0.17 (95% CI -0.02, 0.36) 7 studies, I squared 1.2%, RE. Favours intervention	0	0	NA: all eligible studies reported on this outcome	5

*: number of unpublished studies required to overturn review conclusions after allowing for ORB. When the result is statistically significant this implies the result becoming non-significant. When the result is not statistically significant, the reported value is the number required to change the direction of treatment effect.

NA: Not Applicable; RE: random effects

## Results from the ORB assessment

### Review assessment

The review inclusion criteria stated that trials that reported a measure of pulmonary function or hospital admission as an outcome were eligible. There were 24 studies included in the review. Of these, 22 studies reported on pulmonary function and 18 studies reported on hospital admission.

The review stated that two studies [17,18] were excluded: one study [18] because it did not report either of these outcome measures and the other [17] because it was only available in abstract form and the authors could not be contacted.

Therefore in total there were four studies [17-20] that did not report pulmonary function and eight studies that did not report hospital admission [17,18,21-26]. Since both outcomes are important clinical outcomes in this area [14], the robustness of the conclusions in the review due to selective reporting should be considered.

### Trial assessment and classifications

The outcome matrix (Table 1) shows which outcomes were reported for each trial differentiating between those which were fully reported or partially reported, i.e. effect size or precision only reported along with sample size or a *p-*value [27]. This helps in the assessment of ORB in some cases, for example it can be seen that length of hospital stay was fully reported for one study and yet hospital admission was not reported [18] raising suspicions that ORB may have occurred in this trial.

Four of the authors [KD, RKD, CP, SM - two clinicians and two statisticians] gave a classification independently to each eligible study that did not report hospital admission or pulmonary function. The individual and overall classifications with reasons are given in Additional file 1. The differences were discussed between the four authors and an overall classification agreed. Consensus was easily reached for the majority of study classifications. However, for the abstract by Dadhich *et al. *[17] it was more difficult to agree on an overall classification for pulmonary function due to the information reported.

### Information obtained from trialists

Lead authors were contacted first by email and if no reply was received, then by post. If no contact details could be located or no reply was received, co authors were contacted. There was an attempt to contact all trialists of the studies that did not report on the review outcomes of interest. Responses were received from nine out of the ten trialists [18-26], either lead or co authors. Additional file 1 shows the responses received and it is assumed the information given by trialists regarding whether or not the outcome was measured is correct.

### Pulmonary function

Four studies did not report pulmonary function. Contact with the authors confirmed that two of the studies [18,20] not reporting pulmonary function did not measure it. The authors of one study [19] sent the summary data for this outcome and the *p-value *indicated that it was not statistically significant. Our assessment of the classifications of ORB were correct in these three cases (75%). There was no reply from one author [17]. Without additional information for this study we are unable to confirm whether the classification is correct however the suspicion of ORB remains high.

### Hospital admission

Eight studies did not report hospital admission. There was no reply from one author [17]. For three studies [18,22,25] all patients were hospitalised over the study period hence the outcome was not applicable due to the study design but this was not clear in the trial report. One study [23] reported length of hospital stay. Their correspondence suggests they reported hospital admission as ER admits, however, the study inclusion criteria requires admission to ER. Further clarification was sought but not provided. One of the remaining three studies [21] not reporting hospital admission did measure this outcome but found no difference between the groups. Two studies did not measure hospital admission [24,26]. Our assessment was therefore correct in only one of these eight cases [21] which was mainly due to the outcome, hospital admission being harder to define compared to pulmonary function.

## Results of the Sensitivity analysis for assessing the impact of ORB

The results of the sensitivity analysis are provided in Table 3. Although there was a high level of suspicion of ORB for the study by Dadhich *et al. *[17] it is not included in the sensitivity analysis; although the abstract states that they used nebulised magnesium it does not state whether the trial was conducted in children or adults. Therefore it is not known which meta-analysis it would have been included in had relevant results been reported.

### Intravenous magnesium sulphate

For intravenous magnesium sulphate compared with placebo, the review concluded that "it is an effective treatment in children, being associated with a significant improvement in pulmonary function and a 30% decrease in hospital admissions." However, they found "weak evidence that intravenous magnesium sulphate improves pulmonary function in adults, but no evidence of a significant effect upon hospital admissions, although the data do not exclude a potential reduction in admissions of up to 30%."

**Figure 1:**
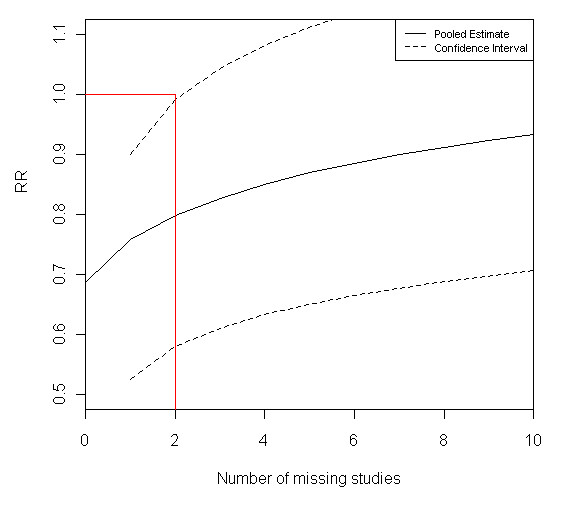
**Plot of the treatment effect estimate using the bound for maximum bias sensitivity analysis for hospital admission for children**.

The sensitivity analysis indicated that the conclusions of the review would not be overturned (i.e. when the result is statistically significant, the conclusions would be overturned if the result becomes non-significant. When the result is not statistically significant, the conclusions would be overturned if the direction of treatment effect changed). However, for hospital admission in children, after allowing for the study suspected of ORB it was estimated that the results may not be robust if one further unpublished study was identified (Appendix 3 and Figure 1). As results were supplied for one study, the meta-analysis for pulmonary function in adults was updated (Table 3).

### Nebulised magnesium sulphate

For nebulised magnesium sulphate, the review concluded that "insufficient data exist to draw reliable conclusions regarding the role of nebulised magnesium sulphate in children" but found "weak evidence that nebulised magnesium sulphate improves pulmonary function and reduces hospital admissions in adults." The results of the sensitivity analysis indicated that the conclusions of the review would not be overturned, changing the direction of the treatment effect (Table 3).

## Discussion

Systematic reviewers should routinely assess the potential impact of ORB. Cochrane systematic reviewers are now recommended to undertake an assessment of this risk of bias. This tutorial demonstrates how to apply a recently developed nine point classification system to the trials included in a systematic review to assess ORB and when a meta-analysis is conducted, how to conduct a sensitivity analysis to consider the robustness of results to both ORB and study publication bias. Examining the existence and potential impact of these biases will increase the validity of inferences concerning healthcare interventions.

### Strengths and limitations of the study

The authors who conducted the review '*Intravenous and nebulised magnesium sulphate for acute asthma' *did request unpublished data from the trialists by email with little success. This was due to both difficulties in locating trialists and their co authors since many trials were quite old, and a lack of response. There has been more success during this work in contacting trialists, through searching for more recent published work by authors to obtain up to date contact details, by contacting colleagues of authors and attempting to contact all co authors on the published papers.

A limitation of this work is that while Cochrane reviews include a list of excluded studies together with reasons for exclusion, the vast majority of reviews, which are non-Cochrane reviews (80%) [28] often do not. If a review does not state any studies as excluded, the review authors should be contacted to ask if any studies were excluded and the reasons for exclusion. If it proves impossible to obtain this information, a thorough assessment of ORB cannot be completed until the review is updated and the search strategy re run.

There was a large amount of heterogeneity between studies, possibly due to the different measures used to assess pulmonary function (e.g. FER and PEFR). Heterogeneity needs to be investigated through meta-regression, sub group analysis, or ideally an individual patient data meta-analysis. The values of *I *squared [29] shown in Table 3 (70.6%, 84.4%, 88.9%) raise the question as to whether a meta-analysis should have been conducted. However, even when there is a large amount of heterogeneity, it may be preferable to quantitatively synthesise the data rather than a qualitative interpretation of the results, as long as the limitations of the methods are properly acknowledged [30].

Classifications G and H are subjective and therefore it is recommended that this assessment should be completed by two experienced reviewers independently and differences discussed. Further data on interrater variation is required, to investigate how much this depends on the review context and the degree of clinical experience. Statisticians are used to thinking about the potential for bias so it may be helpful to include them in this process. It may be easier to predict classifications for some outcomes compared to others. In the example used in this paper, pulmonary function was easier to predict as it is a measure used in clinical practice whilst hospital admission is used more for research and was more difficult to predict. Health care provision and facilities vary in different countries. Hospital admission is likely to have been measured in most studies but some may not have wanted to use it as an outcome measure since it is open to many more confounding factors, compared with pulmonary function tests (PFTs). Hospital admission is a weak surrogate for response to treatment, since numerous other considerations enter into the decision to admit: treating physician bias, type of insurance (e.g USA), home environment and ability to obtain medication plus known or suspected compliance [31]. Further examples of the different classifications are shown in Table 1 of the supplementary material in the main ORBIT paper [10].

The sensitivity analysis is computationally simple and therefore quick to compute and can be estimated for any type of outcome. Work has been undertaken to show the accuracy of the classification system [10] and the robustness of this method of sensitivity analysis [8].

### Conclusions and policy implications for systematic reviews

These methods are useful for those conducting systematic reviews and explains the steps in assessing a review for ORB. Trials with missing outcome data should be scrutinised by reviewers, and a trialist contacted if a study does not report results for the outcomes of interest. The lack of reporting of specified outcome(s) should not be an automatic reason for exclusion of studies. Methods that have been developed to assess the robustness of the conclusions of systematic reviews to ORB should be applied.

A balance needs to be considered between the use of resources and the quality of a review. For example, if results from several studies are missing, (whether the results are pooled or discussed narratively) the review may be of high risk of bias and incorrect conclusions regarding treatment may be made. This is especially important to consider when there is a large proportion of participants in eligible studies which have not been included in the meta-analysis compared to those that are included in the meta-analysis [32]. Another important aspect to consider is when the outcome of interest is one that would be expected to be commonly collected by most investigations of a particular intervention.

Approximately half of all reviews do not report a meta-analysis [28]. It is important to know how to assess ORB when there is no meta-analysis. During the work on a review, data is extracted for each paper and whether or not a meta-analysis is conducted, each outcome is summarised. Therefore, it should be possible to tell which studies do not report the outcomes of interest and still apply the nine point classification system and assess the risk of bias.

Trialists are sometimes contacted during the review process for other information regarding study design and therefore it is suggested that trialists should also be asked about outcomes. It has been shown that it is possible to contact authors for older trials and obtain information and even data, if one persists. This should be encouraged in order to maximise the validity of a review. We found that a Google search often led to more recent contact details.

This work is important in raising awareness of the problem of outcome reporting bias and the importance of assessing ORB within a review and corresponding trial reports. Clinical trials registers and the advance publication of detailed protocols with an explicit description of outcomes and analysis plans should help combat these problems, along with the development of core outcomes for specific clinical areas [33]. Legitimate changes to outcomes stated in the protocol, protocol amendments, and the statistical analysis plans should be described by trialists in the manuscript. Trialists should be encouraged to write up and submit for publication without selection of results, especially with the increased use of online journals where more space is available.

### Future research

There are other outcomes which are important in asthma, such as intubation and the need for ICU, intravenous therapy and an asthma severity score (there are over 15). These outcomes are measured and reported in a variable way. It has been suggested that the development of a set of universally agreed outcomes for a condition could improve the quality of trials and help reduce the selective reporting of outcomes [33,34]. Therefore the development of a set of core outcomes and measurement scales for asthma is crucial.

In the meantime, this work provides guidance to those conducting systematic reviews and by following the steps described here, the risk of outcome reporting bias within a review can be assessed and its potential impact on the results and conclusions discussed.

## Competing interests

The authors declare that they have no competing interests.

## Authors' contributions

KD, CG and PRW contributed to the development of the idea for this work. SM responded to initial queries and gave permission to use their review. KD contacted all authors of included studies and wrote the first draft. KD, RKD, SM and CP classified each of the eligible trials which did not report on one or both outcomes. CG, PRW, RKD, SM and CP provided comments on the manuscript. All authors approved the final manuscript.

## Appendix

### Appendix 1: Summary of the ORBIT study

The ORBIT study examined the prevalence of outcome reporting bias in RCTs and its impact on a large, unselected cohort of Cochrane reviews.

A nine point classification system for missing outcome data in randomised trials was developed and applied to the trials within these Cochrane reviews.

Outcome reporting bias was suspected in at least one trial in more than a third of reviews.

In a sensitivity analysis, nearly a fifth of statistically significant meta-analyses of the review primary outcome would have become non-significant after adjusting for outcome reporting bias and a quarter would have overestimated the treatment effect by 20% or more.

Outcome reporting bias is an under-recognised problem that affects the conclusions in a substantial proportion of Cochrane reviews.

### Appendix 2: How to assess a review for outcome reporting bias

**Step 1**: Check the reasons why studies were excluded from the review to ensure no eligible studies are excluded only because they do not report the outcome of interest. If this information is not available, contact the review authors for information.

**Step 2**: Check to see if any of the included studies do not report on the outcomes of interest.

**Step 3**: Obtain the trial reports for those studies that do not report the outcome of interest or were excluded for not reporting the outcomes of interest. Construct an outcome matrix to indicate which review outcomes were reported in the trial reports, along with other trial outcomes.

**Step 4**: Decide on a classification (Table 2) for each study that does not report the outcome of interest and give a reason for the chosen classification to indicate your level of suspicion of ORB.

**Step 5**: Contact trial authors to find out if they did measure the outcome of interest (the truth) and try to obtain the data (aggregate results or individual patient data) to include in an update of the review. If data are unavailable, ask why they did not report the outcome of interest.

However, when conducting a review, step 5 should be attempted before step 4.

**Step 6**: Conduct a sensitivity analysis to assess the robustness of the conclusions of the review after taking into account the trialists response for studies where it is suspected outcome reporting bias has occurred i.e. for classifications A, D, E and G (high risk of ORB) unless in the case of a G classification it is thought that the outcome was probably not reported because there were no events. The sensitivity analysis can also be extended to assess the effect of unpublished studies that are not known about due to study publication bias.

### Appendix 3: Sensitivity analysis example

#### Intravenous magnesium, children - Hospital admission

The bias bound is obtained by calculating the bias *b *and then adding (or subtracting) this value to the pooled treatment effect estimate on a symmetric scale to move it closer to the null.

To calculate the bound (*b*), values are needed for *n *(the number of trials included in the meta-analysis), *m *(the number of studies in which there is a high suspicion of ORB plus a range of values for the potential number of unpublished trials), σ_*i*_(the standard errors of the trials included in the meta-analysis) and τ^*2*^(the between study variance). In this case, *n *= 3 and *m *= 1 (for ORB) and 2 to 10 (for example, for study publication bias) after taking into account information from trialists (Additional file 1). The values for σ_*i *_and τ^*2 *^are shown in Additional file 2.

The values are then inserted into equation for *b*, when m = 1.

The value b is added to the original pooled treatment effect estimate (Figure 1) to move the result towards the null. For this outcome, the estimate used was the relative risk (RR). Therefore, the value of *b *is added to the log of the pooled relative risk. The same is also done for the lower and upper bound of the confidence intervals.

The adjusted estimate (RR 0.76 (95% CI 0.58, 0.99)) is less favourable towards magnesium sulphate than the original meta-analysis however the result is still statistically significant. This implies that the conclusion of the review for the effect of intravenous magnesium on hospital admission in children appears to be robust to ORB. The red lines in Figure 1 indicate that it would take results from one further study to be missing (either the outcome not being reported in a published study or the whole study being unpublished) to change the results from being statistically significant to non-significant (i.e. for the upper confidence interval to cross one indicating no significant difference). Figure 1 shows how the adjusted pooled estimate and confidence intervals change as the number of missing studies increases.

## Supplementary Material

Additional file 1 Classifications comparison table. Click here for file

Additional file 2 Hospital admission: data for included studies. Click here for file
