# Supplementary material for: Assessing the potential for outcome reporting bias in a review: a tutorial
Source: Trials. 2010 May 12;11:52. doi: 10.1186/1745-6215-11-52 (PMC2888817; doi:10.1186/1745-6215-11-52)
Supplement: Additional file 1 — Classifications comparison table. [file 1745-6215-11-52-S1.DOC]

**Table 4**: Classifications comparison table

| **Outcome** | **Study** | **Classifications*** | | | | **Overall classification** | **Comments** | **Information obtained from the trialists** |
| --- | --- | --- | --- | --- | --- | --- | --- | --- |
|  |  | **1** | **2** | **3** | **4** |  |  |  |
| Pulmonary function | Dadhich, 2003 [17] | **A** | **B** | **A** | **A/B** | **E** | Information from ABSTRACT only (article NOT published)  The abstract states: “Parameters measured PEFR, FEV1, FVC, FEV1/FVC at baseline, 10 min and 20 min interval along with vital parameters and side effects. Observed an increase in all parameters (PEFR, FEV1, FVC, FEV1/FVC) but insignificant (p>0.05). However, mean % increase over baseline was quite significant (p<0.01) at 10 & 20 min interval in group B & C in whom MgSO4 was used. MgSO4 induced greater bronchodilation in those patients having baseline PEFR < 50% [ASA=acute severe asthma] in contrast to salbutamol.”  Reasons for choice of classification: The first part of this paragraph states clearly that the outcome was measured but is not comparing the groups. The last sentence is only for a subgroup of patients. This raises our suspicion of ORB. | The trialist has been emailed for data on pulmonary function but there has been no reply. |
|  | Santana, 2001 [18] | **H** | **G** | **G** | **H** | **H** | Enrolled children 2-12 years (mean 4.5 years). The trialists use other respiratory measurements: breaths / min, SaO2, blood gases, number nebulisations, O2 therapy.  Reason for choice of classification: Patients with exacerbations are often monitored with PEFR to assess severity, so although not stated, we envisage this has probably been measured in older children who are able to manage accurate measurements with PEFR. However, this measurement is **not** applicable to the whole population studied. As this is not applicable to the whole population, it may have not been used to compare patients.  This outcome was reported in the Gurkan and two Ciarollo studies as these studies only included children from the age of six. Also, even though the Devi study included children from the age of one, they only looked at this outcome in children from the age of five years old. | The authors stated “We have to inform that we did not measure the pulmonary function of the patients at the admission to the hospital (study entry), neither during the time the spent in the hospital. We did not measure the pulmonary function because the aim of our study was verify the clinical impact of these interventions (oxygen requirement, increasing the interval between the beta2 agonist nebulization and others). As this study was conducted inside of a Pediatric Emergencey Department, the pulmonary function is not available at every time conducting in such studies.”  Therefore the outcome was not measured in this study. |
|  | Boonyavorakul, 2000 [19] | **E** | **E** | **C** | **E** | **E** | The trial report states that the FISCHL index is measured, which includes: pulse rate, respiratory rate, paradoxical pulse, ***PEFR*,** dypsnoea, accessory muscle use, wheezing. PEFR was measured with Mini-Wright peak flow meter and the highest 3 values were recorded. Data was collected at baseline, 60, 120,180 and 240 mins. Hence pulmonary function was clearly recorded but only reported in the form of the FISCHL index. A graph of the FISCHL index score versus time was also presented.  Reason for choice of classification: The outcome was obviously measured but not necessarily analysed alone as it was part of the FISCHL index, so it is structurally related to this outcome and it is also a commonly reported outcome in asthma. | Authors **s**ent pulmonary function (peak expiratory flow rate) data for both groups; treatment mean 201 (114.6), control mean 189.1 (79.6) along with the *p-value,* *p*=0.66. |
|  | Scarfone, 2000 [20] | **H** | **G** | **G** | **H** | **H** | The trial report states that the degree of improvement is assessed by pulmonary index score. Pulmonary index score includes: respiratory rate, wheezing, inspiratory/expiratory ratio, accessory muscles and oxygen saturation. The study included patients with moderate – severe asthma; children 1-**18** years (children)  This study did not report on pulmonary function but did report on hospital admission (Relative Risk (RR) 0.86, 95% CI: 0.50, 1.49). Here, we would be less suspicious that selective reporting had occurred as although the outcome that was reported favoured magnesium it was not statistically significant.  Reason for choice of classification: Study population included children of all ages. PEFR is likely to have been measured clinically in the older children but less appropriate (hence pulmonary index score) in the younger ones. Patients with exacerbations are often monitored with PEFR to assess severity, so although not stated, envisage this has probably been measured in older children who are able to manage accurate measurements with PEFR. However, this measurement **not** applicable to the whole population studied. As this is not applicable to the whole population, it may have not been used to compare patients. | The authors stated “We did not assess pulmonary function tests as an outcome measure in our study. We had found in previous work that young children in respiratory distress were not able to reliably perform portable spirometry correctly.”  Therefore the outcome was not measured in this study. |
| Hospital admission | Dadhich, 2003 [17] | **G** | **H** | **G** | **H** | **G** | Information from ABSTRACT only (article NOT published)  No mention of hospital admission implied or otherwise.  Reason for choice of classification: Hospital admission is likely to have been measured in most studies (generally in A&E admissions are recorded anyway, so it would not be difficult to have access to this data during analysis). However some trialists may not have wanted to use it as an outcome measure since it is open to many more confounding factors, compared with pulmonary function tests. Hospital admission is a weak surrogate for response to treatment, since numerous other considerations enter into the decision to admit:   - - Treating physician bias   - Type of insurance (e.g. USA)   - Home environment   - Ability to obtain medication + known or suspected compliance [35]   On balance it was felt likely to have been measured and analysed. | The trialist has been emailed for data on hospital admission but there has been no reply. |
|  | Santana, 2001 [18] | **E** | **E** | **E** | **E** | **E** | The outcome is measured and gives the number of patients (and %) admitted to each of the different units. However, overall results are quoted therefore one is unable to distinguish the results for the Magnesium and control groups.  Results states: “no significant difference in relation to hospital stay of the study groups.” Gives results for length of stay in hospital for the study groups (mean and SD) and respective place of stay and results accompanied by p values  Inclusion criteria: patients were admitted to the observation ward of the hospital.  Methods: we followed up hospitalization of each patient without influencing therapeutics or medical prescriptions.  Reason for choice of classification: as results for treatment groups are not presented and the overall values are only given this would be a high suspicion classification as they did measure the outcome. | The authors stated that “all children were admitted to the hospital. It should be considered that the study was performed in the observational area (7 boxes) inside of the emergency department. In this area, the rule is that between 12 and 24 hours the patients should be discharged or transferred to the pediatric wards (including PICU). All these children were admitted to the hospital ward. It is our policy in the Pediatric department discharging or transferring to the pediatric ward all children that stay more than 24 hours in that unit. Aside of this fact, considering the severity of the patients and the interventions that were “new” at that time (infusion of Beta agonist or magnesium sulfate) we decided to maintain these children in the hospital for close observation. In this aspect, the best outcome would be admission to the PICU! The LOS as well as the admission rate to the general pediatric ward have the above described bias.” |
|  | Bijani, 2002 [22] | **D** | **D** | **G** | **H** | **G** | Followed up for 6 hours.  The trial report states in the discussion: “finally our data also supported previous studies (references given) that MgSO4 is helpful for decrease of asthma complications. It seems that administration of MgSO4 in addition to improvement of pulmonary function and helpfulness in the treatment of our patients with acute non-responding asthma can decrease admission rate in patients with acute severe asthma.”  Reason for d classification: The sentence above depends on your interpretation as to whether they are discussing previous studies (as referenced) or referring to their own. Originally we thought the final sentence referred to the trial report but after discussion we now think that they are referring to the other studies discussed above.  Reason for choice of classification: Hospital admission is likely to have been measured in most studies (generally in A&E admissions are recorded anyway, so it would not be difficult to have access to this data during analysis, but not sure if the system is the same in Iran) but some may not have wanted to use it as an outcome measure since it is open to many more confounding factors, compared with PFTs. | The authors stated “We treated asthmatic crisis and gave MG sulphate during acute attacks. We admitted patients at hospital during the trial. After improvement of asthmatic crisis they continued conventional therapy. However they did not compare the rate of readmission in patients with and without previous Mg sulphate therapy for subsequent effect of Mg sulphate in reducing admission rate. Our conclusion was not documented by follow up study of the treated patients but only based on our hypothesis. All patients were admitted and hospitalized in hospital over the study period. All patients stayed at least for two days after recovery of their acute attacks. Overall duration of hospital stay was less than one week about 5 days (average).”  It was not stated in the paper that all patients were admitted to hospital as part of the study design. |
|  | Bessmertny, 2002 [21] | **G** | **G** | **G** | **H** | **G** | Inclusion criteria: patients presenting to ED. Followed up for 2 hours  No mention of hospital admission implied or otherwise.  Reason for choice of classification: Hospital admission is likely to have been measured in most studies (generally in A&E admissions are recorded anyway, so it would not be difficult to have access to this data during analysis). Some may not have wanted to use it as an outcome measure since it is open to many more confounding factors, compared with PFTs.  On balance it was felt likely to have been measured and analysed. | The authors stated “We did measure hospital admission. I will have to go back to our data and see what the results were. If memory serves, there was no difference between the groups and not enough power to make a conclusion about it.” |
|  | Gurkan, 1999 [24] | **G** | **G** | **G** | **H** | **G** | Followed up for 90 minutes.  Intensive care admissions measured. Hence, it is more likely that hospital admissions (in general) were also measured but may not have been analysed.  Reason for choice of classification: Hospital admission is likely to have been measured in most studies (generally in A&E admissions are recorded anyway, so it would not be difficult to have access to this data during analysis but not sure if the system is the same in Turkey). Some may not have wanted to use it as an outcome measure since it is open to many more confounding factors, compared with PFTs.  On balance it was felt likely to have been measured and analysed. | The authors stated “We did not look at hospital admission.” |
|  | Devi, 1997 [23] | **D** | **G** | **G** | **H** | **G** | The trial report specifically states: criteria for discharge, length of hospital stay in hours (Mg = 13.6+/-6.8, control = 18.9 +/- 7.7; p< 0.05) - presumably in the hospital paediatric ER.  Inclusion criteria: admitted to ER. Monitored until discharge from ER  Discussion states: “our data are consistent with reports of recent studies which have shown improvement in lung functions (ref quoted) and decrease in hospitalization (ref quoted) in adults with severe asthma who received IV MgSO4.”  Reason for choice of classification: Hospital admission is likely to have been measured in most studies (generally in A&E admissions are recorded anyway, so it would not be difficult to have access to this data during analysis). Some may not have wanted to use it as an outcome measure since it is open to many more confounding factors, compared with PFTs.  The statement in the discussion section makes us think that hospital admission may have been recorded and analysed, as they state their data is consistent with other studies with decreased hospital admission.  On balance it was felt likely to have been measured and analysed. | The authors stated “The study we did was in a very busy centre with limited PICU and ward beds. Many of the times we admit to ER and continue treatment until we had a bed available. So we had to report it as ER admits.” |
|  | Tiffany, 1993 [26] | **G** | **G** | **G** | **H** | **G** | No mention of hospital admission implied or otherwise.  Inclusion criteria included patients presented to the emergency department. Patients followed up for 5 hours.  Reason for choice of classification: Hospital admission is likely to have been measured in most studies (generally in A&E admissions are recorded anyway, so it would not be difficult to have access to this data during analysis). Some may not have wanted to use it as an outcome measure since it is open to many more confounding factors, compared with PFTs.  On balance it was felt likely to have been measured and analysed. | The authors stated “There is no admission data from that data set.    We did not control hospital admission (decision left to the treating physician) and did not collect that information.  We did not retain patient identifiers after the study, and given the length of time elapsed since it was performed you cannot hope to reconstruct the admissions and discharges.” |
|  | Meral, 1996 [25] | **G** | **G** | **G** | **H** | **G** | No mention of hospital admission implied or otherwise. Patients were followed up for 6 hours  Reason for choice of classification: Hospital admission is likely to have been measured in most studies (generally in A&E admissions are recorded anyway, so it would not be difficult to have access to this data during analysis but not sure if the system is the same in Turkey). Some may not have wanted to use it as an outcome measure since it is open to many more confounding factors, compared with PFTs.  On balance it was felt likely to have been measured and analysed. | The author stated “Children were hospitalised in short stay day care units and were followed up at least 6 hours there. The patients were observed for 24 hours if the symptoms persisted they were taken to a ward.”  The trialist has been emailed for data on hospital admission but there has been no reply. |

* The numbers 1-4 represent the four authors who assessed the trial reports for ORB.
