# Supplementary material for: Assessing the potential for outcome reporting bias in a review: a tutorial
Source: Trials. 2010 May 12;11:52. doi: 10.1186/1745-6215-11-52 (PMC2888817; doi:10.1186/1745-6215-11-52)
Supplement: Additional file 2 — Hospital admission: data for included studies. [file 1745-6215-11-52-S2.DOC]

Table 5: Hospital admission: data for included studies.

| **Study** | **a** | **n1** | **c** | **n2** | **1/a** | **1/n1** | **1/c** | **1/n2** |  |  |  |  |  |
| --- | --- | --- | --- | --- | --- | --- | --- | --- | --- | --- | --- | --- | --- |
| **Ciarallo 1996**  **[35]** | 11 | 15 | 16 | 16 | 0.091 | 0.067 | 0.063 | 0.063 | 0.024 | 0.011 | 0.035 | 5.345 | 28.571 |
| **Ciarallo 2000 [36]** | 8 | 16 | 14 | 14 | 0.125 | 0.063 | 0.071 | 0.071 | 0.063 | 0.011 | 0.074 | 3.676 | 13.514 |
| **Scarfone 2000 [20]** | 11 | 24 | 16 | 30 | 0.091 | 0.042 | 0.063 | 0.033 | 0.078 | 0.011 | 0.089 | 3.352 | 11.236 |
|  |  |  |  |  |  |  |  |  |  |  | **SUM** | **12.373** | **53.321** |

**a** is the number of events in the treatment group

**n1** is the sample size of the treatment group

**c** is the number of events in the control group

**n2** is the sample size of the control group
